# Risk of ulceration in people with diabetes mellitus and factors associated with adherence to self-care

**DOI:** 10.1590/0034-7167-2024-0626

**Published:** 2026-08-17

**Authors:** Danielle Mendonça Oliveira, Giovanna Lanna Araújo Carvalho, Laura Andrade Pinto, Thallita Cláudia Moraes Barbosa, Andreza Oliveira-Cortez, Manuela de Mendonça Figueirêdo Coelho, Juliano Teixeira Moraes, Daniel Nogueira Cortez

**Affiliations:** I Universidade Federal de São João del-Rei. Divinópolis, Minas Gerais, Brazil; II Universidade de Itaúna. Itaúna, Minas Gerais, Brazil; III Universidade Federal do Ceará. Fortaleza, Ceará, Brazil

**Keywords:** Diabetic Foot, Diabetes *Mellitus*, Self Care, Risk Factors, Primary Health Care, Pie Diabético, Diabetes *Mellitus*, Autocuidado, Factores de Riesgo, Atención Primaria de Salud.

## Abstract

**Objectives:**

to analyze sociodemographic, clinical, and ulceration risk factors associated with adherence to self-care in people with diabetes *mellitus* in Primary Health Care.

**Methods:**

this cross-sectional study included 769 people with diabetes *mellitus* using the Diabetes Self-Care Activities Questionnaire. Univariate analysis and confirmatory factor analysis were employed to verify the associations.

**Results:**

44.1% of participants presented a risk for foot ulceration. Factor analysis indicates that blood sugar testing and medication adherence have the highest factor loadings (λ=0.76 and λ=0.54, respectively) in adherence to self-care for diabetes *mellitus*. The number of appointments, diagnosis time, and polypharmacy were associated with blood sugar testing and medication adherence.

**Conclusions:**

adherence to self-care remains centered on biomedical practices. More than half of the sample presented a risk for foot ulceration, which reinforces the need for more effective preventive actions in Primary Care.

## RESUMEN

**Objetivos:**

analizar los factores de riesgo sociodemográficos, clínicos y de úlceras asociados con la adherencia al autocuidado en personas con diabetes *mellitus* en Atención Primaria de Salud.

**Métodos:**

estudio transversal con 769 personas con diabetes *mellitus*, utilizando el Cuestionario de Actividades de Autocuidado de la Diabetes. Se utilizaron análisis univariados y análisis factorial confirmatorio para verificar las asociaciones.

**Resultados:**

el 44,1% de los participantes presentó riesgo de ulceración del pie. El análisis factorial indica que el control de la glucemia y el uso de medicamentos presentan la mayor carga factorial (λ=0,76 y λ=0,54, respectivamente) en la adherencia al autocuidado de la diabetes *mellitus*. El número de consultas, el tiempo de diagnóstico y la polifarmacia se asociaron con la monitorización de la glucemia y el uso de medicamentos.

**Conclusiones:**

la adherencia al autocuidado sigue centrándose en las prácticas biomédicas. Más de la mitad de la muestra presentó riesgo de ulceración del pie, lo que refuerza la necesidad de acciones preventivas más eficaces en Atención Primaria.

## INTRODUCTION

Brazil ranks 6^th^ in the world in prevalence of diabetes *mellitus* (DM), totaling 15.7 million adults (20 to 79 years old) with the diagnosis^(1)^.

DM is a public health problem, especially due to the high rates of complications^(2)^. People with good glycemic control can prevent or delay the onset of complications, such as diabetes *mellitus*-related foot disease (DFD), when they adhere to self-care practices such as proper treatment and lifestyle changes^(3,4)^. It should be noted that the term “diabetic foot” was replaced by “DFD”, according to the International Working Group on the Diabetic Foot, in 2023^(4)^.

In Brazil, it is expected that follow-up, prevention, and intervention services will be offered primarily by the Primary Health Care (PHC) multidisciplinary team. Family Health Strategy (FHS) nurses play a fundamental role, in which, through nursing appointments and actions involving health education, they can identify risk factors for foot ulcers and guide patients on self-care practices, with the aim of preventing complications related to DM^(5,6)^. Self-care is an important practice for DM prevention and control, as well as being fundamental for adherence to treatment and reduction of its complications, since it integrates the ability to care for oneself and provides the performance of activities essential to achieving, maintaining, or promoting a healthy life^(3)^. Adherence to self-care activities is related to personal, cultural, and socioeconomic factors, as well as aspects related to the disease, treatment, the health system, and the multidisciplinary team. The conditioning factors that influence the quality of self-care practices are age, sex, culture, religion, education, and social and economic vulnerability^(7,8)^.

Given this context, research focused on self-care and DM is related to glycemic control, but there is a gap in self-care analysis when it comes to the risk of foot ulcers^(3,5,8)^. In this regard, one of the necessary actions in PHC is to classify the risk in order to define the care and prevention actions for the progression of alterations so that the chance of success in these actions depends on the self-care that a person with DM demonstrates regarding their feet and legs^(3,5,6)^.

Therefore, considering that DM is a multifactorial disease, it is essential that nurses are able to identify which factors most interfere with self-care, in order to design a more assertive therapeutic plan that will have a significant impact on adherence to practices that promote self-care and, consequently, reduce complications, especially DFD^(6,7)^.

## OBJECTIVES

To analyze sociodemographic, clinical, and ulceration risk factors associated with adherence to self-care in people with DM in PHC.

## METHODS

### Ethical aspects

The study was conducted in accordance with national and international ethical guidelines and approved by the *Universidade Federal de São João del-Rei* Research Ethics Committee, Midwest Campus. All participants signed the Informed Consent Form.

### Study design, period, and location

This is a cross-sectional study conducted with people with DM from PHC in a municipality in Minas Gerais, following the STrengthening the Reporting of OBservational studies in Epidemiology framework. The study was carried out in urban FHSs between June 2022 and January 2023.

### Sample, inclusion and exclusion criteria

The Municipal Health Department of this municipality initially provided the number of patients attended by FHSs. An eligible population of 4,826 people with DM registered in urban FHS units was considered. To calculate the sample size, the finite population formula n/(1 + (n-1)/N) was used, with a significance level of 95% and a margin of error of 2.5%. Considering that the studies did not present a mean adherence to self-care, 50% of the population proportion was used, arriving at a minimum sample size of 768 people with DM. Through stratified sampling by FHS, a total of 769 participants was obtained.

To select individuals with DM for the sample, 15 of the 32 FHSs were randomly selected for the study. For each FHS, stratification was performed based on the number of individuals with DM. All individuals with DM registered at the FHSs participated in the study, excluding those under 18 years of age and those unable to answer the questionnaires.

### Study protocol

The response or dependent variable was adherence to self-care, and the explanatory or independent variables were age, sex, marital status, education level, occupation, Body Mass Index, duration of DM, number of appointments in the last two years, foot examination by a healthcare professional, polypharmacy, use of psychotropic drugs, and risk of foot ulceration.

The study variables were defined, *a priori,* by the group of researchers, who are stomatherapists and were responsible for data collection and assessment of people with DM. Training for data collection took place after training and pilot testing.

The identification of potential participants occurred through appointments to the municipal health information system and collaboration with community health workers. Participants were contacted by telephone, welcomed, and informed about the purpose of the contact and the research. Data collection was then scheduled according to their availability. Data collection took place in person at the health units to which participants were affiliated, always after reading, clarifying doubts, and signing the Informed Consent Form.

The collection of sociodemographic and clinical variables was carried out using standardized questionnaires designed in a structured manner. For adherence to self-care, the Summary of Diabetes Self-Care Activities (SDSCA), validated and translated into Portuguese for Brazil, was used^(9)^.

The SDSCA has six dimensions and 15 items: “general diet” (two items), “specific diet” (three items), “physical activity” (two items), “blood sugar testing” (two items), “foot care” (three items), and “medication adherence” (three items, used according to the medication regimen). For each item, the number of times a participant performed the activity in question over the last seven days is quantified, with 0 being the least desirable situation and 7 the most favorable. In the specific diet items that ask about the consumption of foods high in fat and sweets, the values were reversed (if 7 = 0, 6 = 1, 5 = 2, 4 = 3, 3 = 4, 2 = 5, 1 = 6, 0 = 7 and vice versa). Finally, for each dimension, the mean adherence to self-care reported in the previous week was calculated. The total SDSCA score results from the sum of the scores of the six dimensions, with a possible variation between 0 and 105. Higher scores indicate greater adherence to self-care. There are no universally established cut-off points for classifying adherence; median values or adapted criteria are used according to the research context^(9)^.

Polypharmacy was defined as the use of five or more medications, according to the latest update^(10)^. Foot ulceration risk classification was carried out, according to the 2023 consensus^(4)^, after performing a physical examination of the feet and legs, in addition to identifying or not identifying peripheral arterial disease (PAD) and loss of protective sensation (LPS). According to the consensus, PAD is defined by an ankle-brachial index (ABI) value less than 0.9 or greater than 1.3. The ABI was obtained using the MEDPEJ^®^ vascular doppler (approved by the Brazilian National Health Regulatory Agency), dividing the dorsalis pedis or posterior tibial artery systolic blood pressure measurement by the brachial systolic blood pressure measurement.

To assess LPS, a 128 Hz tuning fork and a 10-gram Semmes-Weinstein monofilament (orange) were used. Vibratory sensitivity was assessed with a 128 Hz tuning fork. Protective tactile sensitivity was assessed with a Semmes-Weinstein monofilament (10 grams), with pressure maintained for 1-2 seconds in a quiet place with a comfortable room temperature. LPS was considered present when there was an absence of vibratory sensitivity to the tuning fork placed on the dorsal surface of the distal phalanx of the first toe (hallux) and/or absence of tactile sensitivity to the touch of the monofilament on the posterior surface of the hallux, head of the first and fifth metatarsals^(4)^.

For the risk of ulceration, the following was stratified as risk 0 (very low): absence of PAD or LPS; risk 1 (low): presence of PAD or LPS; risk 2 (moderate): association of PAD and LPS or PAD plus foot deformity or LPS plus foot deformity; and risk 3 (high): occurrence of PAD or LPS associated with a history of previous ulcer or amputation of lower limbs or end-stage renal disease^(4)^. It is noteworthy that ulceration is the most complex final stage in DFD progression, whether or not it is accompanied by amputation.

### Analysis of results and statistics

To describe participants’ profile, absolute and relative frequencies of qualitative variables were calculated, as well as the median and interquartile range of quantitative variables. For the results of self-care adherence indices, data normality was tested using the Shapiro-Wilk test. Subsequently, the Mann-Whitney test was performed to assess the association between the risk of foot ulceration and associated factors. The level of statistical significance established was 5% (p ≤ 0.05).

A confirmatory factor analysis (CFA) was conducted to verify the SDSCA factor structure adequacy to the investigated sample, as well as to identify which factor has the highest factor loading, i.e., which best explains the self-care activities of the studied sample. This type of analysis is widely used to assess whether the factor organization of a proposed model is in accordance with the theoretical foundations that support the construct. Unlike exploratory approaches, CFA requires that the factor structure be previously defined by the researcher, since its purpose is essentially confirmatory. CFA was incorporated into the study after the initial stage of analyzing SDSCA scores, with the aim of verifying the adequacy of the instrument’s factor structure in the investigated population. This decision was based on the need to ensure that the original configuration of the questionnaire remained valid in the specific context of PHC, considering the particularities of participants’ profile. CFA application contributed to reinforcing the validity of the results obtained and directing data interpretation, focusing on the most representative dimensions of self-care. It is important to note that CFA was complementary and exploratory in nature, without the intention of proposing a new factor structure for the instrument.

Parameter estimation was performed using the Robust Diagonally Weighted Least Squares method, which is appropriate for categorical data. To determine the model fit, the following indices were considered: χ^2^, χ^2^/df; Comparative Fit Index (CFI); Tucker-Lewis Index (TLI); Standardized Root Mean Residual (SRMR); and Root Mean Square Error of Approximation (RMSEA). It is expected that the χ^2^ value will not be significant, that the χ^2^/df ratio will be less than 5, preferably less than 3, and that CFI and TLI will exceed 0.90, with ideal values above 0.95. Furthermore, the RMSEA should be less than 0.08, ideally below 0.06, with the upper limit of the confidence interval less than 0.10. For factor loadings, values greater than 0.40 were considered acceptable.

Although the SDSCA score shows a discrete distribution, multivariate linear regression was chosen because the dependent variable exhibits continuous behavior with adequate amplitude and moderate asymmetrical distribution. Furthermore, the linear model assumptions were verified and considered met for the study’s objectives.

The data generated during the process were organized into spreadsheets in Microsoft Excel and subsequently exported to the Statistical Package for the Social Sciences version 23.0 for descriptive, inferential, and correlational analyses. CFA, in turn, was conducted using the JASP.

## RESULTS

Of the 769 participants in this study, the majority were female (65.5%), aged 60 or older (59.9%), with a partner (58.3%), with incomplete primary education (61.2%), and not employed (73%). Concerning clinical characteristics, most people had a DM diagnosis time ≤ ten years (51.2%) and used polypharmacy (73.7%). It was also found that 47.7% had more than four appointments for DM follow-up in the last two years; and 77.4% reported that they had not had their feet examined by a healthcare professional in the last two years.

The median SDSCA score was 17.66. This value represents a low level of adherence, especially considering the maximum possible score of 105 points. The instrument scores were correlated with sociodemographic and clinical variables and are presented in Table 1.

**Table 1:** Association of sociodemographic and clinical variables and Summary of Diabetes Self-Care Activities score, Divinópolis, Minas Gerais, Brazil, 2023

Variables	Median	U	Z	p^*^	r^α^
Sociodemographic variables		69808.000	-0.393	0.694	
Age					
<60 years (n=308; 40.1%)	17.50				
> = 60 years (n=461; 59.9%)	17.83				
Sex		62832.500	-1.349	0.177	
Male (n=265 34.5%)	18.16				
Female (n=504 65.5%)	17.33				
Education		69136.000	-0.348	0.728	
>= High school (n=471; 65.5%)	17.66				
< High school (n=298; 38.8%)	17.66				
Income		72864.500	-0.281	0.778	
> 02 minimum wages (n=365; 41.7%)	17.33				
<02 minimum wages (n=404; 52.5%)	17.91				
Occupation		53999.000	-1.588	0.112	
Active (n=208; 27%)	16.83				
Inactive (n=561; 73%)	18.16				
Marital status		69566.500	-0.770	0.442	
Companion (n=448; 58.3%)	18.00				
No companion (n=321; 41.7%)	17.33				
Race		64543.500	-0.558	0.577	
White (n=260; 33.8%)	17.83				
Non-white (n=509; 66.2%)	17.66				
Clinical variables					
Appointments		63284.500	-3.407	**0.001**	0.12
>= 04 (n=367; 47.7%)	18.83				
<04 (n=402; 52.3%)	16.66				
DM classification		2784.500	-3.370	**0.001**	0.12
DM 1 (n=15; 2%)	22.66				
DM 2 (n=754; 98%)	17.50				
Diagnosis time		55347.000	-6.018	**0.000**	0.22
<= 10 years (n=394; 51.2%)	16.33				
> 10 years (n=375; 48.8%)	19.66				
Polypharmacy		48009.500	-3.415	**0.001**	0.12
Yes (n=567; 73.7%)	18.33				
No (n=202; 26.3%)	16.66				
Psychotropic		46550.000	-0.780	0.436	
Yes (n=159; 20.7%)	17.66				
No (n=610; 79.3%)	17.66				
Suspected PAD		68524.000	-0.984	0.325	
No suspicion (n= 454; 59%)	17.50				
Suspected (n= 315; 41%)	18.00				
Loss of protective sensation		28781.000	-0.896	0.370	
Present (n=90; 11.7%)	18.41				
Absent (n=679; 88.3%)	17.50				
Foot examined by a professional		37545.000	-5.518	**0.000**	0.20
Yes (n=174; 22.6%)	19.75				
No (n=595; 77.4%)	17.00				
Poor foot hygiene		21055.000	-1.065	0.287	
Yes (n=65; 8.5%)	17.16				
No (n= 704; 91.5%)	17.83				
BMI		61771.000	-1.121	0.262	
Normal weight (n=251; 32.6%)	17.83				
Overweight/Obese (n=518; 67.4%)	17.50				

* *Teste de Mann-Whitney; αEffect size; DM - diabetes mellitus; PAD - peripheral arterial disease; BMI - Body Mass Index.*

*SDSCA - Summary of Diabetes Self-Care Activities; SDSCAGD - general diet; SDSCASD - specific diet; SDSCPA - physical activity; SDSCBST - blood sugar testing; SDSCFC - foot care; SDSCAMA - medication adherence.*

Although the variables related to the number of appointments made, DM classification, diagnosis time, polypharmacy, and having feet assessed by healthcare professionals showed significance, the effect size of all of them was considered small (<0.50). It was found that 44.1% (n=339) of participants presented a risk of foot ulceration.

When we performed regression analysis to understand if the overall SDSCA score is associated with the risk of foot ulceration, no significant association was found (p=0.157). However, considering the six different factors of the SDSCA that take into account diet, physical activity, blood sugar testing, foot care, and medication adherence, we opted to analyze the scale factors and the various variables that can predict the risk of foot ulceration.

**Figure 1:**
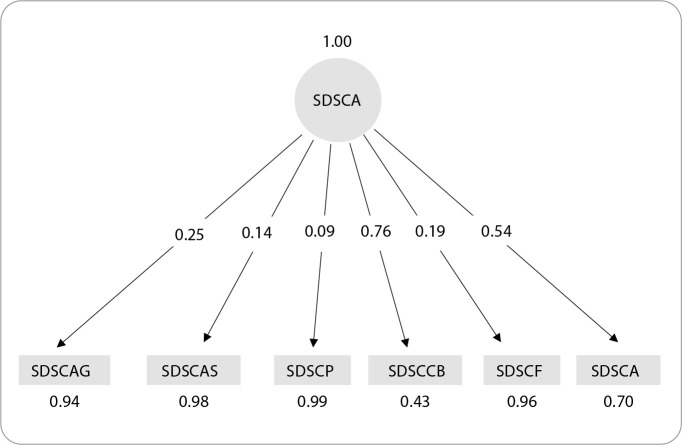
Factor model for analysis of the Summary of Diabetes Self-Care Activities (Primary Health Care), Divinópolis, Minas Gerais, Brazil, 2023

The SDSCA’s CFA was performed on this population to verify the plausibility of the instrument’s structures in the studied sample. This structure presented contradictory fit results. As can be seen in Table 2, chi-square values were significant, and the chi-square to degrees of freedom ratio was somewhat high (5.47), with p<0.001. CFI (0.838) and TLI (0.730) did not support the model; however, RMSEA (0.076, minimum-maximum 0.056 -0.098) and SRMR (0.05) values were satisfactory. Thus, since this study did not aim to propose a new factorial model for the scale used, but to identify which factors have the most influence on the SDSCA construct, factorial model 01 was maintained, as shown in Figure 1.

**Table 2:** Regression model of sociodemographic and clinical variables in relation to the blood sugar testing dimension of the Summary of Diabetes Self-Care Activities, Divinópolis, Minas Gerais, Brazil, 2023

(Constant)			Standardized coefficient		
	t	Sig	Beta	Min.	Max.
	6.407	0.000		8.394	15.809
**Sociodemographic predictors**					
Age	-3.288	**0.001**	-0.146	-0.052	-0.013
Sex	-2.065	**0.039**	-0.075	-0.791	-0.020
Education	-0.672	0.502	-0.025	-0.521	0.255
Income	-0.490	0.624	-0.018	-0.457	0.274
Occupation	1.008	0.314	0.041	-0.226	0.704
Marital status	0.922	0.357	0.033	-0.195	0.541
Race	-0.195	0.846	-0.007	-0.400	0.328
**Clinical predictors**					
Number of appointments	-4.168	**0.000**	-0.144	-1.095	-0.394
DM classification	-4.047	**0.000**	-0.149	-4.136	-1.434
Diagnosis time	4.795	**0.000**	0.171	0.522	1.246
Polypharmacy	-2.488	**0.013**	-0.092	-0.968	-0.114
Psychotropic use	1.266	0.221	0.043	-0.165	0.715
Suspected PAD	0.740	0.460	0.025	-0.217	0.481
Loss of protective sensation	-1.478	0.140	-0.051	-0.949	0.134
Foot examined by a professional	-3.398	**0.001**	-0.118	-1.150	-0.308
Poor foot hygiene	-0.519	0.604	-0.018	-0.793	0.461
BMI	-0.563	0.573	-0.020	-0.043	0.024

*DM - diabetes mellitus; PAD - peripheral arterial disease; BMI - Body Mass Index; Min. - minimum; Max. - maximum.*

Factor analysis indicates that blood sugar testing and medication adherence have the highest factor loadings (λ= 0.76 and λ= 0.54, respectively) and, consequently, a greater influence on the SDSCA results. Thus, a multivariate analysis of sociodemographic and clinical data impacting the risk of foot ulceration was performed using the two factors of the scale, in order to understand which variables are significant and how they interfere with medication adherence and blood sugar testing.

Although CFA did not demonstrate an ideal fit for CFI and TLI, RMSEA and SRMR values were considered satisfactory. Principal component analyses were not conducted, as the focus was on testing the previously established theoretical structure of SDSCA.

Table 2 presents the multivariate analysis of sociodemographic and clinical variables in relation to the blood sugar testing factor.

The model was significant (F (17, 46, 21) = 785.627, p=0.000). The results demonstrated the influence of sociodemographic and clinical characteristics on blood sugar testing scores, as a construct to measure self-care in people with DM with a significant model (F (17, 8.000) = 785.627, p=0.000).

Regarding blood sugar testing scores, age is a significant predictor: for each year of life, there is a 0.146-point reduction in the score. Women have 0.075 standard deviations (SD) lower scores than men, and people with fewer than four annual appointments have 0.144 SD lower scores than those who have four or more appointments. Individuals with more than ten years of diagnosis have 0.177 SD more than those with a diagnosis of ten years or less. People who do not use polypharmacy have 0.092 SD lower blood sugar testing scores than those who use polypharmacy. Similarly, those who do not have their feet examined by healthcare professionals have 0.118 SD lower scores than those who do.

Table 3 presents the multivariate analysis of sociodemographic and clinical variables in relation to the medication adherence factor.

**Table 3:** Regression model of sociodemographic and clinical variables in relation to the medication adherence dimension of the Summary of Diabetes Self-Care Activities, Divinópolis, Minas Gerais, Brazil, 2023

(Constant)			Standardized coefficient		
	t	Sig	Beta	Min.	Max.
	5.193	0.000		4.118	9.123
**Sociodemographic predictors**					
Age	-1.374	0.170	-0.063	-0.022	0.004
Sex	-0.490	0.624	-0.018	-0.325	0.195
Education	-2.517	**0.012**	-0.096	-0.598	-0.074
Income	0.745	0.456	0.028	-0.153	0.341
Occupation	-0.199	0.842	-0.008	-0.346	0.282
Marital status	-0.488	0.625	-0.018	-0.310	0.187
Race	-0.137	0.891	-0.005	-0.263	0.229
**Clinical predictors**					
Number of appointments	-2.704	**0.007**	-0.096	-0.563	-0.089
DM classification	0.431	0.667	0.016	-0.712	1.112
Diagnosis time	4.537	**0.000**	0.166	0.320	0.809
Polypharmacy	-4.424	**0.000**	-0.168	-0.937	-0.361
Psychotropic use	2.131	**0.033**	0.077	0.025	0.620
Suspected PAD	0.779	0.436	0.027	-0.142	0.329
Protective sensation in the feet	-1.062	0.289	-0.037	-0.563	0.168
Foot examined by a healthcare professional	-2.711	0.007	-0.097	-0.677	-0.108
Poor foot hygiene	-1.082	0.280	-0.038	-0.657	0.190
BMI	1.214	0.225	0.045	-0.009	0.037

*DM - diabetes mellitus; PAD - peripheral arterial disease; BMI - Body Mass Index; Min. - minimum; Max. - maximum.*

This analysis also demonstrated the influence of sociodemographic and clinical characteristics on medication adherence scores as a constituent factor for self-care in people with DM, with a significant model (F (17, 5,548) = 248.222, p=0.000).

Here, the number of appointments, diagnosis time, and polypharmacy were also significant variables, indicating that those who had fewer than four appointments per year had 0.096 SD fewer scores, those with more than ten years of diagnosis had 0.166 SD more scores than those with less time, and individuals who do not use polypharmacy had 0.168 SD fewer scores.

People with less than a high school diploma have 0.096 SD fewer scores than people with high school or higher education, and participants who do not use psychotropic drugs have 0.077 SD more in scores related to medication adherence.

## DISCUSSION

The results of this study reveal a pattern of adherence to self-care centered mainly on biomedical practices, such as blood sugar testing and medication adherence, while behavioral and preventive dimensions, such as diet, physical activity, and foot care, showed low adherence. This trend highlights the predominance of a care model still oriented by prescriptive and curative logic, which goes against the comprehensive proposal of PHC, whose mission includes empowering the user through health education and encouraging active self-care.

Among the clinical factors analyzed, diagnosis time, number of appointments, polypharmacy, and foot assessment by healthcare professionals showed an association with greater adherence to biomedical dimensions. This indicates that as a person lives longer with the diagnosis and engages more frequently with healthcare services, they tend to adhere more rigorously to therapeutic recommendations, especially those related to glycemic and medication control. However, the effect sizes were small, which points to a limited explanatory power of the analyzed variables on adherence levels, suggesting the need to include contextual, psychosocial, and behavioral variables in future analyses.

The biomedical model, still present in healthcare services, contributes to the fragmentation of care by prioritizing prescriptive interventions focused on medication adherence as the main therapeutic strategy^(11)^. As a result, self-care practices that require behavioral change, such as healthy eating, regular physical activity, and foot care, remain undervalued and rarely incorporated into patients’ routines^(9,12-14)^. In this context, the monitoring carried out by nurses, especially in PHC, represents a strategic opportunity to overcome this fragmented logic. Through a patient-centered approach, nurses have the potential to promote comprehensive care and strengthen patient autonomy, contributing to adherence to practices that go beyond the medication-based control of the disease^(11,12)^. In this sense, a study conducted in an outpatient clinic focused on the care of DFD demonstrated that consultations performed by nurses promoted positive changes in self-care practices, especially related to adopting a healthy diet and engaging in physical exercise, in addition to preventive skin and nail care during daily foot examinations^(8)^.

Non-pharmacological practices, such as physical activity, healthy eating, and foot care, showed low adherence rates, highlighting weaknesses in self-care management among people with DM. This scenario reveals the complexity involved in promoting behavioral changes, recognized as one of the greatest challenges in DM management. Adherence to a dietary plan, for instance, is often perceived as a restrictive and punitive process, rather than a nutritional re-education strategy, which compromises its implementation^(9)^. Similarly, lack of motivation, disinterest, and contextual limitations are significant barriers to the regular practice of physical activity^(10,12)^. A systematic review indicated that social issues, such as support from others, better access, and shorter travel distances, were the main barriers and facilitators for engaging in physical activity among the Brazilian population^(10)^.

From a sociodemographic perspective, advanced age and female sex were associated with lower adherence to blood sugar testing. Although this practice is fundamental for self-care, many individuals with DM, especially older adults, have difficulty adhering to it. Factors such as the high costs of monitoring systems, the need for maintenance with frequent sensor replacements, and physical and cognitive limitations, such as loss of manual dexterity and decline in cognitive function, contribute to this barrier^(13)^. In this context, when analyzing the usability and adoption of health technology devices among the elderly, it is important to consider these individual, psychological, and physical factors^(14)^. Assessments should encompass not only technological effectiveness, but also older adults’ ability to integrate these devices into their daily self-care^(15)^.

The results demonstrated that women presented significantly lower scores regarding blood sugar testing. This finding can be attributed to several factors, including work overload and the triple burden faced by many of them. This situation aligns with the literature, which discusses socially imposed gender responsibilities that often lead women to prioritize the care of others at the expense of their own health needs. The fear of being seen as selfish or negligent when choosing to dedicate limited time and resources to self-management of DM highlights the social pressure these women face^(16)^. Thus, the study analysis emphasizes the need for interventions that take into account the social and family context of women with DM. It is essential to promote inclusive self-care strategies that offer emotional and practical support, aiming to improve adherence to blood sugar testing and disease control.

A significant association was observed between polypharmacy use and greater adherence to blood sugar testing. Individuals using multiple medications appear to be more attentive to glucose control, likely due to the need to manage drug interactions or the greater severity of their disease. This relationship suggests that, even with the complexity of multipharmacy treatment, these individuals tend to be more committed to therapeutic recommendations, possibly as a coping strategy to better manage their health condition^(6,7)^. In contrast to these findings, a cross-sectional study with 321 people with DM in Indonesia identified that those with polypharmacy had poor glycemic control and low adherence to physical activity^(17)^.

Treatment adherence is also influenced by the time of diagnosis. Individuals with shorter diagnosis times tend to have lower adherence scores, which may indicate a phase of denial or the perception that the disease does not yet represent a real threat. However, the literature indicates that the time of diagnosis can significantly impact the emotions of people with DM, with attitudes tending to become more positive over the years^(18)^. Nurses play a crucial role in raising awareness among these patients from the outset about the complications of DM and the importance of early and continuous treatment^(19)^. This association, however, should not be interpreted as a direct causal relationship, but possibly as a reflection of a learning and adaptation process that develops over time, as the person comes to cope with the demands of treatment and recognize the risks associated with the disease.

Another key finding was that individuals with fewer than four annual appointments, whether with a physician or nurse, showed lower adherence to self-care, highlighting the need for proactive outreach by healthcare professionals. A lack of regular appointments can be detrimental to the prevention of serious complications, such as those related to DM. Early detection of foot problems is crucial to minimizing complications, as well as reducing morbidity and mortality related to DFD^(20)^. However, many people only seek help when complications have already developed. This highlights the importance of primary prevention, which should include a consistent routine of preventative foot care^(21)^.

Participants with more than four appointments in the last two years for DM follow-up did not undergo foot assessment by a healthcare professional. This gap highlights a weakness in the care offered for DM. It is crucial to emphasize that, as indicated in the literature, foot care receives limited attention compared to other complications associated with DM^(4,22)^.

People with lower levels of education also presented lower scores in the medication adherence dimension, which may be explained by the difficulty in understanding medical and pharmacological instructions. The level of education was identified as the main variable associated with self-care regarding DFD in a meta-analysis that evaluated predictors for self-care practices^(23)^. Diabetes education increases knowledge and skills, enabling people to better understand their condition and become active participants in their treatment. Understanding diabetes treatment and management, as well as risk factors and complications, increases self-efficacy and improves self-management behavior^(24)^.

Another important finding of the study was that patients who do not use psychotropic drugs showed greater adherence to medication treatment. This suggests that patients’ mental and emotional state may influence their ability to correctly follow treatment^(25)^. Therefore, it is important for nurses to pay attention to these issues and, if necessary, involve mental health professionals in the care of patients who need additional support^(26)^.

Taken together, these results highlight the complexity of self-care for DM, indicating the need for personalized and educational approaches throughout treatment.

The results presented have practical implications for healthcare professionals, who should adopt an educational, accessible, and proactive approach to improve patient adherence to self-care. This includes the use of technologies adapted for different audiences, actively seeking out patients with less frequent appointments, and teaching strategies that make treatment more understandable and feasible for everyone. These measures are essential to prevent complications such as DFD and improve the quality of life of people with DM.

Although some variables showed a statistically significant association with SDSCA scores, the observed effect sizes were small (r<0.50), suggesting that such associations may not have great clinical relevance. In this sense, the identified factors may explain a fraction of the variability in self-care adherence scores. Therefore, it is important to interpret the results with caution and consider future analyses that include other contextual, behavioral, or psychosocial variables that may contribute more significantly to understanding self-care adherence in DM.

### Study limitations

Among the study’s limitations are the fact that it is cross-sectional, which does not allow establishing a cause-and-effect relationship between adherence to self-care and explanatory variables, especially regarding the risk of ulceration; the selection of participants only from the urban FHS, excluding other spaces where patients with DM are treated in PHC; and the relative generalization of the results, since, although the municipality where the data was collected has a considerable population, it is not possible to extend them to other Brazilian municipalities.

Another study limitation relates to the absence of contextual and psychosocial variables, such as support network, access to health services, and family support, which directly influence adherence to self-care. Furthermore, specific assessments of participants’ cognition or emotional state were not included, which restricts the interpretation of findings such as the use of psychotropic drugs.

### Contributions to nursing and health

The study highlights findings that raise concerns about DM and the need for PHC to include a focus on the legs and feet. For many years, there have been manuals and guidelines on PHC, emphasizing the FHS as the entry point and responsible for DM control, including the prevention of complications and/or early detection. However, the increase in ulcerations and amputations resulting from DFD raises concerns about this public health problem^(4,27)^. Nurses can be key professionals for screening for foot ulcers in people with DM.

## CONCLUSIONS

The analyses indicated that clinical factors, such as diagnosis time, number of appointments, and polypharmacy, influence self-care scores. However, the small effect sizes suggest that sociodemographic and contextual variables, such as emotional support, support network, and access to services, also play a relevant role and deserve to be explored in future studies.

The study identified that almost half of the sample presented a risk for foot ulceration according to clinical criteria for LPS and/or PAD, which reinforces the need for more effective preventive actions in PHC. Furthermore, adherence to self-care among people with DM in PHC remains centered on biomedical practices, such as medication adherence and blood sugar testing, while activities requiring behavioral change, such as foot care, diet, and physical activity, show low adherence.

Hence, it becomes urgent to strengthen multidisciplinary actions in PHC, with an emphasis on continuous and personalized educational approaches capable of promoting patient autonomy and the integration of care focused on the prevention of complications, such as DFD.

## Data Availability

The research data are available only upon request.
