# Supplementary material for: Risk of ulceration in people with diabetes mellitus and factors associated with adherence to self-care
Source: Rev Bras Enferm. 2026 Aug 17;79:e20240626. doi: 10.1590/0034-7167-2024-0626 (PMC13481041; doi:10.1590/0034-7167-2024-0626)
Supplement: Translation

# Translation: Risco de ulceração em pessoas com diabetes *mellitus* e fatores associados à adesão ao autocuidado

**DOI:** 10.1590/0034-7167-2024-0626pt

**Authors:** Danielle Mendonça Oliveira, Giovanna Lanna Araújo Carvalho, Laura Andrade Pinto, Thallita Cláudia Moraes Barbosa, Andreza Oliveira-Cortez, Manuela de Mendonça Figueirêdo Coelho, Juliano Teixeira Moraes, Daniel Nogueira Cortez

**Affiliations:** I Universidade Federal de São João del-Rei. Divinópolis, Minas Gerais, Brasil; II Universidade de Itaúna. Itaúna, Minas Gerais, Brasil; III Universidade Federal do Ceará. Fortaleza, Ceará, Brasil

**Keywords:** Pé, Diabético, Diabetes *Mellitus*, Autocuidado, Fatores de Risco, Atenção Primária à, Saúde.

## Abstract

**Objetivos:**

analisar os fatores sociodemográficos, clínicos e risco de ulceração associados à adesão ao autocuidado em pessoas com diabetes *mellitus* na Atenção Primária à Saúde.

**Métodos:**

estudo transversal com 769 pessoas com diabetes *mellitus* utilizando o Questionário Atividades de Autocuidado com o Diabetes. Empregaram-se análise univariada e análise fatorial confirmatória para verificar as associações.

**Resultados:**

44,1% dos participantes apresentaram risco para ulceração dos pés. A análise fatorial aponta que os fatores monitorização da glicemia e uso de medicamentos possuem maior carga fatorial (λ=0,76 e λ=0,54, respectivamente) na adesão ao autocuidado para o diabetes *mellitus*. O número de consultas, tempo de diagnóstico e polifarmácia apresentaram associação com a monitorização da glicemia e uso de medicamentos.

**Conclusões:**

a adesão ao autocuidado permanece centrada em práticas biomédicas. Mais da metade da amostra apresentou risco para ulceração nos pés, o que reforça a necessidade de ações preventivas mais eficazes na Atenção Primária.

## INTRODUÇÃO

O Brasil ocupa o 6º lugar em prevalência de diabetes *mellitus* (DM) no mundo, totalizando 15,7 milhões de pessoas adultas (20 a 79 anos) com o diagnóstico^(1)^.

O DM é um problema de saúde pública, especialmente em decorrência das altas taxas de complicações^(2)^. As pessoas com bom controle glicêmico conseguem prevenir ou retardar o aparecimento de complicações, como o surgimento da doença do pé relacionada ao diabetes *mellitus* (DPDM), quando aderem às práticas de autocuidado como realizar o tratamento correto e fazer alterações de comportamento no estilo de vida^(3,4)^. Ressalta-se que o termo “pé diabético” foi substituído por “DPDM”, de acordo com o *International Working Group on the Diabetic Foot*, em 2023^(4)^.

No Brasil, espera-se que os serviços de acompanhamento, prevenção e as intervenções sejam ofertados, principalmente, pela equipe multidisciplinar da Atenção Primária à Saúde (APS). O enfermeiro da Estratégia Saúde da Família (ESF) tem um papel fundamental, no qual, por meio da consulta de enfermagem e de ações que envolvem a educação em saúde, pode identificar os fatores de risco de ulceração nos pés e direcionar o paciente sobre as práticas de autocuidado, com o objetivo de prevenção das complicações que estão relacionadas ao DM^(5,6)^. O autocuidado é uma prática importante para a prevenção e controle do DM, além de fundamental para a adesão ao tratamento e redução de suas complicações, já que ele integra a capacidade de cuidar de si e proporciona o desempenho de atividades indispensáveis para alcançar, manter ou promover uma vida saudável^(3)^. A adesão às atividades de autocuidado está relacionada a fatores de ordem pessoal, cultural e socioeconômica, além de aspectos relativos à doença, ao tratamento, ao sistema de saúde e à equipe multiprofissional. Já os fatores condicionantes que influenciam a qualidade das práticas do autocuidado são idade, sexo, cultura, religião, escolaridade, e vulnerabilidade social e econômica^(7,8)^.

Diante deste contexto, as investigações voltadas ao autocuidado e o DM se relacionam ao controle glicêmico, mas há uma lacuna para a análise do autocuidado quando se trata do risco de ulceração nos pés^(3,5,8)^. Neste sentido, uma das ações necessárias na APS é classificar o risco para delimitar as ações de cuidado e prevenção do avanço das alterações, de modo que a chance de sucesso nessas ações passe pelo autocuidado que a pessoa com DM apresenta quanto aos pés e pernas^(3,5,6)^.

Portanto, considerando que o DM é uma doença multifatorial, é fundamental que os enfermeiros consigam identificar quais os fatores que mais interferem no autocuidado, de forma a delinear plano terapêutico mais assertivo para que realmente haja impacto significativo na adesão de práticas que favoreçam o autocuidado e, com isso, haja redução de complicações, em especial a DPDM^(6,7)^.

## OBJETIVOS

Analisar os fatores sociodemográficos, clínicos e risco de ulceração associados à adesão ao autocuidado em pessoas com DM na APS.

## MÉTODOS

### Aspectos éticos

O estudo foi conduzido de acordo com as diretrizes de ética nacionais e internacionais, e aprovado pelo Comitê de Ética em Pesquisa da Universidade Federal de São João del-Rei, *Campus* Centro-Oeste. Todos os participantes assinaram o Termo de Consentimento Livre e Esclarecido.

### Desenho, período e local do estudo

Trata-se de estudo transversal realizado com pessoas com DM da APS de um município de Minas Gerais, seguindo o referencial *STrengthening the Reporting of OBservational studies in Epidemiology*. O estudo foi realizado nas ESFs urbanas entre junho de 2022 e janeiro de 2023.

### Amostra, critérios de inclusão e exclusão

A Secretaria Municipal de Saúde deste município disponibilizou, inicialmente, o número de pacientes atendidos pelas ESFs. Considerou-se uma população elegível de 4.826 pessoas com DM cadastradas nas unidades de ESFs urbanas. Para calcular o tamanho da amostra, utilizou-se a fórmula de população finita n/(1 + (n-1)/N), nível de significância de 95% e margem de erro de 2,5%. Considerando que os estudos não apresentaram média de adesão ao autocuidado, utilizaram-se 50% de proporção da população, chegando-se ao tamanho amostral mínimo de 768 pessoas com DM. Por meio da amostragem estratificada por ESF, obteve-se ao final um total de 769 participantes.

Para a seleção das pessoas com DM que compuseram a amostra, entre as 32 ESFs, foram sorteadas 15 para o estudo. Para cada ESF, realizou-se a estratificação por número de pessoas com DM. Participaram do estudo todas as pessoas com DM adstritas das ESFs, excluindo-se aquelas menores de 18 anos e pessoas que não possuíam condições para responder aos questionários.

### Protocolo do estudo

A variável resposta ou dependente foi adesão ao autocuidado, e as variáveis explicativas ou independentes foram idade, sexo, estado civil, nível de instrução, ocupação, Índice de Massa Corporal, tempo de DM, número de consultas nos últimos dois anos, exame dos pés por um profissional de saúde, polifarmácia, uso de psicotrópicos e risco de ulceração nos pés.

As variáveis do estudo foram definidas, *a priori*, pelo grupo de pesquisadores que são estomaterapeutas e foram responsáveis pela coleta de dados e avaliação das pessoas com DM. O treinamento para a coleta de dados ocorreu após treinamento e realização de testes piloto.

A identificação dos potenciais participantes ocorreu pela consulta ao sistema de informação municipal de saúde e colaboração dos agentes comunitários de saúde. Por meio de ligações telefônicas, os participantes foram contatados, acolhidos e esclarecidos sobre o que se tratava o contato e a pesquisa. Assim, a coleta foi agendada de acordo com a disponibilidade dos mesmos. As coletas ocorreram presencialmente em consultórios das unidades de saúde às quais os participantes eram vinculados, sempre após a leitura, o esclarecimento de dúvidas e a assinatura do Termo de Consentimento Livre e Esclarecido.

A coleta das variáveis sociodemográficas e clínicas foi realizada pela aplicação de questionários padronizados construídos de forma estruturada. Para adesão ao autocuidado, foi utilizado o Questionário de Atividades de Autocuidado com o Diabetes (QAD), validado e traduzido para o Brasil^(9)^.

O QAD apresenta seis dimensões e 15 itens, sendo elas “alimentação geral” (com dois itens), “alimentação específica” (três itens), “atividade física” (dois itens), “monitorização da glicemia” (dois itens), “cuidado com os pés” (três itens) e “uso de medicação” (três itens, utilizados de acordo com o esquema medicamentoso). Para cada item, são quantificadas quantas vezes dos últimos sete dias o participante realizou a atividade questionada, sendo 0 a situação menos desejável, e 7, a mais favorável. Nos itens sobre alimentação específica que perguntam sobre o consumo de alimentos ricos em gordura e doces, os valores foram invertidos (se 7 = 0, 6 = 1, 5 = 2, 4 = 3, 3 = 4, 2 = 5, 1 = 6, 0 = 7 e vice-versa). Ao final, para cada dimensão, foi realizada a média de adesão ao autocuidado referida pela semana anterior. O escore total do QAD resulta da soma dos escores das seis dimensões, com variação possível entre 0 e 105. Escores mais elevados indicam maior adesão ao autocuidado. Não há pontos de corte universalmente estabelecidos para classificação da adesão, sendo utilizados valores medianos ou critérios adaptados conforme o contexto da pesquisa^(9)^.

Considerou-se polifarmácia o uso de cinco ou mais medicamentos, conforme última atualização^(10)^. A classificação de risco de ulceração nos pés ocorreu, conforme consenso de 2023^(4)^, após realização do exame físico dos pés e pernas, além de identificação ou ausência de doença arterial periférica (DAP) e perda da sensibilidade protetora (PSP). De acordo com o consenso, a DAP é definida por valor de índice tornozelo-braço (ITB) menor que 0,9 ou maior que 1,3. O ITB foi obtido por meio da utilização do doppler vascular MEDPEJ^®^ (aprovado pela Agência Nacional de Vigilância Sanitária), com divisão da medida da pressão arterial sistólica da artéria pediosa ou tibial posterior, pela medida da pressão arterial sistólica braquial.

Para avaliação da PSP, foi utilizado diapasão de 128 Hz e monofilamento de Semmes-Weinstein de 10 gramas (laranja). A sensibilidade vibratória foi avaliada com diapasão de 128 Hz. A sensibilidade tátil protetora foi avaliada com monofilamento de Semmes-Weinstein (10 gramas), com pressão mantida por 1-2 segundos em local silencioso e com temperatura ambiente confortável. Considerou-se PSP quando houve ausência de sensibilidade vibratória ao diapasão colocado sobre a face dorsal da falange distal do primeiro pododáctilo (hálux) e/ou ausência de sensibilidade tátil ao toque do monofilamento na face posterior do hálux, cabeça do primeiro e quinto metatarso^(4)^.

Para o risco de ulceração, classificou-se como risco 0 (muito baixo) a ausência de DAP ou PSP; risco 1 (baixo), a presença de DAP ou PSP; risco 2 (moderado), a associação de DAP e PSP ou DAP mais a deformidade nos pés ou PSP mais a deformidade nos pés; e risco 3 (alto), a ocorrência de DAP ou PSP associado à história de úlcera prévia ou amputação de membros inferiores baixos ou estágio final de doença renal^(4)^. Destaca-se que a ulceração é a etapa final mais complexa como desfecho da evolução da DPDM, acompanhada ou não de amputação.

### Análise dos resultados e estatística

Para descrever o perfil dos participantes, foram calculadas as frequências absolutas e relativas das variáveis qualitativas, assim como a mediana e o intervalo interquartil das variáveis quantitativas. Para os resultados dos índices de adesão ao autocuidado, foi realizado o teste de normalidade dos dados utilizando o teste de Shapiro-Wilk. Posteriormente, foi realizado o teste de Mann-Whitney para associação entre o risco de ulceração nos pés e fatores associados. O nível de significância estatística estabelecida foi de 5% (p ≤ 0,05).

Foi conduzida uma análise fatorial confirmatória (AFC) com o objetivo de verificar a adequação da estrutura fatorial do QAD à amostra investigada, bem como apontar qual fator apresenta maior carga fatorial, ou seja, que melhor explique as atividades de autocuidado da amostra estudada. Esse tipo de análise é amplamente utilizado para avaliar se a organização fatorial de um modelo proposto está de acordo com os fundamentos teóricos que sustentam o construto. Diferentemente de abordagens exploratórias, a AFC requer que a estrutura fatorial seja previamente definida pelo pesquisador, uma vez que seu propósito é essencialmente confirmatório. A realização da AFC foi incorporada ao estudo após a etapa inicial de análise dos escores do QAD com o objetivo de verificar a adequação da estrutura fatorial do instrumento na população investigada. Essa decisão se fundamentou na necessidade de assegurar que a configuração original do questionário permanecesse válida no contexto específico da APS, considerando as particularidades do perfil dos participantes. A aplicação da AFC contribuiu para reforçar a validade dos resultados obtidos e direcionar a interpretação dos dados com foco nas dimensões mais representativas do autocuidado. Destaca-se que a AFC teve caráter complementar e exploratório, sem a intenção de propor uma nova estrutura fatorial para o instrumento.

A estimativa dos parâmetros foi realizada utilizando o método *Robust Diagonally Weighted Least Squares*, que é apropriado para dados categóricos. Para determinar o ajuste do modelo, foram considerados os seguintes índices: χ^2^, χ^2^/gl; *Comparative Fit Index* (CFI); *Tucker-Lewis Index* (TLI); *Standardized Root Mean Residual* (SRMR); e *Root Mean Square Error of Approximation* (RMSEA). Espera-se que o valor de χ^2^ não seja significativo, que a razão χ^2^/gl seja inferior a 5, preferencialmente menor que 3, e que os índices CFI e TLI superem 0,90, sendo ideais valores acima de 0,95. Além disso, o RMSEA deve ser inferior a 0,08, idealmente abaixo de 0,06, com o limite superior do intervalo de confiança menor que 0,10. Para as cargas fatoriais, valores superiores a 0,40 foram considerados aceitáveis.

Apesar do escore de o QAD apresentar distribuição discreta, optou-se pelo uso de regressão linear multivariada, uma vez que a variável dependente apresenta comportamento contínuo com amplitude adequada e distribuição assimétrica moderada. Ademais, os pressupostos do modelo linear foram verificados e considerados atendidos para os objetivos do estudo.

Os dados gerados durante o processo foram organizados em planilhas no *Microsoft Excel* e posteriormente exportados para o *software Statistical Package for the Social Sciences*, versão 23.0, para realização de análises descritivas, inferenciais e correlacionais. A AFC, por sua vez, foi conduzida utilizando o *software* JASP.

## RESULTADOS

Dos 769 participantes deste estudo, a maioria era do sexo feminino (65,5%), possuía 60 anos ou mais (59,9%), com companheiro (58,3%), ensino fundamental incompleto (61,2%) e não desempenhava atividade trabalhista (73%). Em relação à caracterização clínica, a maior parte das pessoas apresentava tempo de diagnóstico do DM ≤ dez anos (51,2%) e fazia uso de polifarmácia (73,7%). Verificou-se ainda que 47,7% realizaram mais de quatro consultas para acompanhamento do DM nos últimos dois anos; e 77,4% relataram que não tiveram os pés examinados por um profissional de saúde nos últimos dois anos.

A mediana do QAD foi de 17,66. Esse valor representa uma adesão considerada baixa, especialmente ao se considerar a pontuação máxima possível de 105 pontos. Foi realizada associação dos valores dos escores do instrumento com as variáveis sociodemográficas e clínicas e apresentadas na Tabela 1.

**Tabela 1:** Associação das variáveis sociodemográficas e clínicas e escore do Questionário de Atividades de Autocuidado com o Diabetes, Divinópolis, Minas Gerais, Brasil, 2023

Variáveis	Mediana	U	Z	p^*^	r^α^
Variáveis sociodemográficas		69808.000	-0,393	0,694	
Idade					
<60 anos (n=308; 40,1%)	17,50				
>= 60 anos (n=461; 59,9%)	17,83				
Sexo		62832.500	-1,349	0,177	
Masculino (n=265 34,5%)	18,16				
Feminino (n=504 65,5%)	17,33				
Escolaridade		69136.000	-0,348	0,728	
>= Ensino médio (n=471; 65,5%)	17,66				
<Ensino médio (n=298; 38,8%)	17,66				
Renda		72864.500	-0,281	0,778	
> 02 salários mínimos (n=365; 41,7%)	17,33				
< 02 salários mínimos (n=404; 52,5%)	17,91				
Ocupação		53999.000	-1,588	0,112	
Ativo (n=208; 27%)	16,83				
Inativo (n=561; 73%)	18,16				
Estado civil		69566.500	-0,770	0,442	
Com companheiro (n=448; 58,3%)	18,00				
Sem companheiro (n=321; 41,7%)	17,33				
Raça		64543.500	-0,558	0,577	
Branco (n=260; 33,8%)	17,83				
Não branco (n=509; 66,2%)	17,66				
Variáveis clínicas					
Consultas		63284.500	-3,407	**0,001**	0,12
>= 04 (n=367; 47,7%)	18,83				
<04 (n=402; 52,3%)	16,66				
Classificação do DM		2784.500	-3.370	**0,001**	0,12
DM 1 (n=15; 2%)	22,66				
DM 2 (n=754; 98%)	17,50				
Tempo de diagnóstico		55347.000	-6,018	**0,000**	0,22
<= 10 anos (n=394; 51,2%)	16,33				
> 10 anos (n=375; 48,8%)	19,66				
Polifarmácia		48009.500	-3,415	**0,001**	0,12
Sim (n=567; 73,7%)	18,33				
Não (n=202; 26,3%)	16,66				
Psicotrópico		46550.000	-0,780	0,436	
Sim (n=159; 20,7%)	17,66				
Não (n=610; 79,3%)	17,66				
Suspeita de DAP		68524.000	-0,984	0,325	
Sem suspeita (n= 454; 59%)	17,50				
Com suspeita (n= 315; 41%)	18,00				
Perda da sensibilidade protetora		28781.000	-0,896	0,370	
Presente (n=90; 11,7%)	18,41				
Ausente (n=679; 88,3%)	17,50				
Pés examinados por profissional		37545.000	-5,518	**0,000**	0,20
Sim (n=174; 22,6%)	19,75				
Não (n=595; 77,4%)	17,00				
Má higiene dos pés		21055.000	-1,065	0,287	
Sim (n=65; 8,5%)	17,16				
Não (n= 704; 91,5%)	17,83				
IMC		61771.000	-1,121	0,262	
Eutrófico (n=251; 32,6%)	17,83				
Sobrepeso/obesidade (n=518; 67,4%)	17,50				

* Teste de Mann-Whitney;

α Tamanho de efeito; DM - diabetes mellitus; DAP - doença arterial periférica; IMC - Índice de Massa Corporal.

*QAD - Questionário de Atividades de Autocuidado; QADAD - alimentação geral; QADAE - alimentação específica; QADF - atividade física; QADM - monitorização da glicemia; QADC - cuidado com os pés; QADME - uso de medicação.*

Apesar de as variáveis relacionadas ao número de consultas realizadas, classificação do DM, tempo de diagnóstico, polifarmácia e ter pés avaliados por profissionais de saúde terem apresentado significância, o tamanho de efeito de todos foi considerado pequeno (<0,50). Verificou-se que 44,1% (n=339) dos participantes apresentavam risco de ulceração nos pés.

Ao realizarmos regressão para entender se o escore geral do QAD possui associação com a variável risco de ulceração nos pés, não houve associação significativa (p=0,157). Entretanto, considerando os seis diferentes fatores do QAD que leva em consideração hábitos alimentares, atividade física, mensuração glicêmica, cuidado com os pés e uso de terapia medicamentosa, optou-se por realizar análise dos fatores da escala e as diversas variáveis que podem predizer o risco de ulceração nos pés.

Realizou-se AFC do QAD nessa população de forma a verificar a plausibilidade das estruturas do instrumento na amostra estudada. Tal estrutura apresentou resultados de ajuste contraditórios. Conforme pode ser visto na Tabela 2, os valores de qui-quadrado foram significativos, e a razão qui-quadrado por graus de liberdade foi um pouco elevada (5,47), com p<0,001. CFI (0,838) e TLI (0,730) não suportaram o modelo, entretanto os valores de RMSEA (0,076, mínimo-máximo 0,056 -0,098) e SRMR (0,05) foram satisfatórios. Assim, como neste estudo não se objetivou propor novo modelo fatorial para escala utilizada, mas identificar que fatores possuem mais influência no construto QAD, manteve-se o modelo fatorial 01, conforme apresentado na Figura 1.

**Tabela 2:** Modelo de regressão variáveis sociodemográficas e clínicas em relação à dimensão monitorização da glicemia do Questionário de Atividades de Autocuidado com o Diabetes, Divinópolis, Minas Gerais, Brasil, 2023

(Constante)			Coeficiente padronizado		
	t	Sig	Beta	Mín.	Máx.
	6,407	0,000		8,394	15,809
**Preditores sociodemográficos**					
Idade	-3,288	**0,001**	-0,146	-0,052	-0,013
Sexo	-2,065	**0,039**	-0,075	-0,791	-0,020
Escolaridade	-0,672	0,502	-0,025	-0,521	0,255
Renda	-0,490	0,624	-0,018	-0,457	0,274
Ocupação	1,008	0,314	0,041	-0,226	0,704
Estado civil	0,922	0,357	0,033	-0,195	0,541
Raça	-0,195	0,846	-0,007	-0,400	0,328
**Preditores clínicos**					
Número de consultas	-4,168	**0,000**	-0,144	-1,095	-0,394
Classificação do DM	-4,047	**0,000**	-0,149	-4,136	-1,434
Tempo de diagnóstico	4,795	**0,000**	0,171	0,522	1,246
Polifarmácia	-2,488	**0,013**	-0,092	-0,968	-0,114
Uso psicotrópico	1,266	0,221	0,043	-0,165	0,715
Suspeita de DAP	0,740	0,460	0,025	-0,217	0,481
Perda da sensibilidade protetora	-1,478	0,140	-0,051	-0,949	0,134
Pés examinados por profissional de saúde	-3,398	**0,001**	-0,118	-1,150	-0,308
Má higiene dos pés	-0,519	0,604	-0,018	-0,793	0,461
IMC	-0,563	0,573	-0,020	-0,043	0,024

*DM - diabetes mellitus; DAP - doença arterial periférica; IMC - Índice de Massa Corporal; Mín. - mínimo; Máx. - máximo.*

**Figura 1:** Modelo fatorial para análise do Questionário de Atividades de Autocuidado com o Diabetes (Atenção Primária à Saúde), Divinópolis, Minas Gerais, Brasil, 2023

A análise fatorial aponta que os fatores monitorização da glicemia e uso de medicamentos possuem maior carga fatorial (λ= 0,76 e λ= 0,54, respectivamente) e, consequentemente, maior influência nos resultados do QAD. Assim, realizou-se análise multivariada dos dados sociodemográficos e clínicos que impactam o risco de ulceração nos pés com os dois fatores da escala, de forma a entender quais variáveis são significativas e como interferem no uso de medicamentos e no monitoramento da glicemia.

Apesar de a AFC não ter demonstrado ajuste ideal pelos CFI e TLI, os valores de RMSEA e SRMR foram considerados satisfatórios. Não foram conduzidas análises de componentes principais, visto que o foco foi testar a estrutura teórica previamente estabelecida do QAD.

A Tabela 2 apresenta a análise multivariada das variáveis sociodemográficas e clínicas em relação ao fator monitorização da glicemia.

O modelo foi significativo (F (17, 46,21) = 785.627, p=0,000). Os resultados demonstraram haver influência de características sociodemográficas e clínicas nos escores de medição da glicemia, como construto para mensurar autocuidado de pessoas com DM com modelo significativo (F (17, 8,000) = 785.627, p=0,000).

Quanto aos escores do fator monitorização da glicemia, a idade é um preditor significativo: a cada ano de vida, há uma redução de 0,146 ponto no escore. Mulheres apresentam 0,075 desvio padrão (DP) a menos nos escores do que homens, e pessoas com menos de quatro consultas anuais possuem 0,144 DP a menos nos escores do que as que realizam quatro ou mais consultas. Indivíduos com mais de dez anos de diagnóstico apresentam 0,177 DP a mais do que os que possuem diagnóstico igual ou menor que dez anos. Pessoas que não fazem uso de polifarmácia têm 0,092 DP a menos nos escores de monitoramento da glicemia do que as que fazem uso de polifarmácia. Da mesma forma, as que não têm os pés examinados por profissionais de saúde têm 0,118 DP a menos do que as que têm.

A Tabela 3 apresenta a análise multivariada das variáveis sociodemográficas e clínicas em relação ao fator uso de medicação.

**Tabela 3:** Modelo de regressão variáveis sociodemográficas e clínicas em relação à dimensão uso de medicação do Questionário de Atividades de Autocuidado com o Diabetes, Divinópolis, Minas Gerais, Brasil, 2023

(Constante)			Coeficiente padronizado		
	t	Sig	Beta	Mín.	Máx.
	5,193	0,000		4,118	9,123
**Preditores sociodemográficos**					
Idade	-1,374	0,170	-0,063	-0,022	0,004
Sexo	-0,490	0,624	-0,018	-0,325	0,195
Escolaridade	-2,517	**0,012**	-0,096	-0,598	-0,074
Renda	0,745	0,456	0,028	-0,153	0,341
Ocupação	-0,199	0,842	-0,008	-0,346	0,282
Estado civil	-0,488	0,625	-0,018	-0,310	0,187
Raça	-0,137	0,891	-0,005	-0,263	0,229
**Preditores clínicos**					
Número de consultas	-2,704	**0,007**	-0,096	-0,563	-0,089
Classificação do DM	0,431	0,667	0,016	-0,712	1,112
Tempo de diagnóstico	4,537	**0,000**	0,166	0,320	0,809
Polifarmácia	-4,424	**0,000**	-0,168	-0,937	-0,361
Uso psicotrópico	2,131	**0,033**	0,077	0,025	0,620
Suspeita de DAP	0,779	0,436	0,027	-0,142	0,329
Sensibilidade protetora dos pés	-1,062	0,289	-0,037	-0,563	0,168
Pés examinados por profissional de saúde	-2,711	0,007	-0,097	-0,677	-0,108
Má higiene dos pés	-1,082	0,280	-0,038	-0,657	0,190
IMC	1,214	0,225	0,045	-0,009	0,037

*DM - diabetes mellitus; DAP - doença arterial periférica; IMC - Índice de Massa Corporal; Mín. - mínimo; Máx. - máximo.*

Esta análise também demonstrou haver influência de características sociodemográficas e clínicas nos escores relacionados ao uso de medicação como fator constituinte para autocuidado de pessoas com DM com modelo significativo (F (17, 5,548) = 248.222, p=0,000).

Aqui, número de consultas, tempo de diagnóstico e polifarmácia também foram variáveis significativas, apontando que quem realizou menos de quatro consultas ao ano apresenta menos 0,096 DP nos escores, quem tem mais de dez anos de diagnóstico apresenta 0,166 DP a mais nos escores do que os que possuem menos tempo, e os indivíduos que não fazem uso de polifarmácia têm menos 0,168 DP nos escores.

Pessoas com escolaridade menor que o ensino médio completo apresentam 0,096 DP menos em seus escores do que as pessoas com escolaridade igual ou maior ao ensino médio, e os participantes que não fazem uso de psicotrópicos possuem 0,077 DP a mais nos escores relacionados ao uso de medicação.

## DISCUSSÃO

Os resultados deste estudo revelam um padrão de adesão ao autocuidado centrado majoritariamente em práticas biomédicas, como monitorização da glicemia e uso de medicamentos, enquanto dimensões comportamentais e preventivas, como alimentação, atividade física e cuidados com os pés, apresentaram baixa adesão. Essa tendência evidencia a predominância de um modelo de cuidado ainda orientado pela lógica prescritiva e curativa, o que vai de encontro à proposta integral da APS, cuja missão inclui o empoderamento do usuário por meio da educação em saúde e do incentivo ao autocuidado ativo.

Entre os fatores clínicos analisados, tempo de diagnóstico, número de consultas, polifarmácia e avaliação dos pés por profissionais de saúde demonstraram associação com a maior adesão às dimensões biomédicas. Isso indica que, à medida que a pessoa convive por mais tempo com o diagnóstico e se engaja mais frequentemente com os serviços de saúde, tende a aderir com mais rigor às recomendações terapêuticas, sobretudo aquelas relacionadas ao controle glicêmico e medicamentoso. No entanto, os tamanhos de efeito foram baixos, o que aponta para uma limitada explicabilidade das variáveis analisadas sobre os níveis de adesão, sugerindo a necessidade de incluir variáveis contextuais, psicossociais e comportamentais em análises futuras.

O modelo biomédico ainda presente nos serviços de saúde contribui para a fragmentação do cuidado, ao priorizar intervenções prescritivas centradas no uso de medicamentos como principal estratégia terapêutica^(11)^. Com isso, práticas de autocuidado que exigem mudança de comportamento, como alimentação saudável, atividade física regular e cuidados com os pés, permanecem pouco valorizadas e raramente incorporadas à rotina dos pacientes^(9,12-14)^. Nesse contexto, o acompanhamento realizado por enfermeiros, especialmente na APS, representa uma oportunidade estratégica para a superação dessa lógica fragmentada. Por meio de uma abordagem centrada no sujeito, o enfermeiro tem potencial para promover o cuidado integral e fortalecer a autonomia do paciente, contribuindo para a adesão a práticas que vão além do controle medicamentoso da doença^(11,12)^. Neste sentido, estudo realizado em ambulatório voltado ao cuidado de DPDM demonstrou que as consultas realizadas por enfermeiros promoveram modificações positivas nas ações de autocuidado, especialmente relacionadas à adoção de uma alimentação saudável e à prática de exercícios físicos, além dos cuidados preventivos com a pele e unhas durante o exame diário dos pés^(8)^.

As práticas não farmacológicas, como atividade física, alimentação saudável e cuidados com os pés, apresentaram baixos índices de adesão, evidenciando fragilidades na gestão do autocuidado entre pessoas com DM. Esse cenário revela a complexidade envolvida na promoção de mudanças de comportamento, reconhecidamente um dos maiores desafios no manejo do DM. A adesão a um plano alimentar, por exemplo, é frequentemente percebida como um processo restritivo e punitivo, em vez de uma estratégia de reeducação nutricional, o que compromete sua implementação^(9)^. De modo semelhante, falta de motivação, desinteresse e limitações contextuais figuram como barreiras significativas à prática regular de atividades físicas^(10,12)^. Revisão sistemática apontou que questões sociais, como suporte de outras pessoas, melhor acesso e menor distância de deslocamento, foram as principais barreiras e facilitadores para a realização de atividade física na população brasileira^(10)^.

Do ponto de vista sociodemográfico, a idade avançada e o sexo feminino estiveram associados à menor adesão à monitorização da glicemia. Embora essa prática seja fundamental para o autocuidado, muitos indivíduos com DM, principalmente os idosos, têm dificuldades em aderir a essa prática. Fatores como os altos custos dos sistemas de monitoramento, a necessidade de manutenção com substituições frequentes de sensores e as limitações físicas e cognitivas, como a perda de destreza manual e o declínio da função cognitiva, contribuem para essa barreira^(13)^. Nesse contexto, ao analisar a usabilidade e adesão aos dispositivos tecnológicos de saúde entre os idosos, é importante considerar esses fatores individuais, psicológicos e físicos^(14)^. As avaliações devem abranger não apenas a eficácia tecnológica, mas também a capacidade do idoso em integrar esses dispositivos ao seu autocuidado diário^(15)^.

Os resultados demonstraram que as mulheres apresentaram escores significativamente menores no que diz respeito ao monitoramento da glicemia. Esse achado pode ser atribuído a diversos fatores, incluindo à sobrecarga de trabalho e à tripla jornada enfrentada por muitas delas. Essa situação se alinha com a literatura, que discute as responsabilidades de gênero impostas socialmente que frequentemente levam as mulheres a priorizar o cuidado de outros em detrimento de suas próprias necessidades de saúde. O medo de serem vistas como egoístas ou negligentes, ao escolherem dedicar tempo e recursos limitados à autogestão do DM, evidencia a pressão social que essas mulheres enfrentam^(16)^. Desse modo, a análise do estudo enfatiza a necessidade de intervenções que levem em conta o contexto social e familiar das mulheres com DM. É fundamental promover estratégias de autocuidado inclusivas que ofereçam suporte emocional e prático, visando melhorar a adesão ao monitoramento glicêmico e controle da doença.

Foi observada uma associação significativa entre o uso de polifarmácia e a maior adesão ao monitoramento glicêmico. Indivíduos que utilizam múltiplos medicamentos parecem estar mais atentos ao controle da glicose, provavelmente devido à necessidade de gerir interações medicamentosas ou à maior gravidade da doença. Essa relação sugere que, mesmo diante da complexidade do tratamento com múltiplos medicamentos, essas pessoas tendem a ser mais comprometidas com as recomendações terapêuticas, possivelmente como uma estratégia de enfrentamento para gerenciar melhor sua condição de saúde^(6,7)^. Diferentemente desses achados, estudo transversal com 321 pessoas com DM na Indonésia identificou que aqueles com polifarmácia apresentavam baixo controle glicêmico e baixa adesão para atividade física^(17)^.

A adesão ao tratamento também é influenciada pelo tempo de diagnóstico. Indivíduos com menos tempo de diagnóstico tendem a ter escores menores de adesão, o que pode indicar uma fase de negação ou a percepção de que a doença ainda não representa uma ameaça real. No entanto, a literatura indica que o tempo de diagnóstico pode impactar significativamente as emoções das pessoas com DM, com atitudes que tendem a se tornar mais positivas ao longo dos anos^(18)^. Os enfermeiros têm um papel fundamental em sensibilizar esses pacientes desde o início sobre as complicações do DM e a importância de um tratamento precoce e contínuo^(19)^. Essa associação, contudo, não deve ser interpretada como uma relação causal direta, mas possivelmente como reflexo de um processo de aprendizado e adaptação que se desenvolve ao longo do tempo, à medida que a pessoa passa a lidar com as exigências do tratamento e a reconhecer os riscos relacionados à doença.

Outro ponto de destaque foi que indivíduos com menos de quatro consultas anuais, seja do médico ou do enfermeiro, apresentaram menor adesão ao autocuidado, o que mostra a necessidade de busca ativa por parte dos profissionais de saúde. A falta de consultas regulares pode ser prejudicial na prevenção de complicações graves, como as relacionadas à DPDM. Detectar problemas nos pés de forma precoce é crucial para minimizar complicações, bem como reduzir a morbidade e mortalidade relacionadas ao DM^(20)^. Contudo, muitas pessoas buscam ajuda quando as complicações já estão presentes. Isso ressalta a importância da prevenção primária, que deve incluir uma rotina consistente de cuidados preventivos com os pés^(21)^.

Os participantes com mais de quatro consultas nos últimos dois anos para o acompanhamento DM não foram submetidos à avaliação dos pés por um profissional de saúde. Essa lacuna evidencia uma fragilidade nos cuidados oferecidos para o DM. É crucial salientar que, como indicado na literatura, os cuidados direcionados aos pés recebem uma atenção limitada em comparação com outras complicações associadas ao DM^(4,22)^.

Pessoas com menor nível de escolaridade também apresentaram menores escores na dimensão uso de medicamentos, o que pode ser explicado pela dificuldade em compreender as orientações médicas e farmacológicas. O nível de escolaridade foi apontado como a principal variável associada ao autocuidado com a DPDM em metanálise que avaliou os preditores para as práticas de autocuidado^(23)^. A educação em DM aumenta o conhecimento e as habilidades, permitindo que as pessoas compreendam melhor sua condição e se tornem participantes ativos em seu tratamento. Compreender o tratamento e o gerenciamento do DM, assim como os fatores de risco e suas complicações, aumenta a autoeficácia e melhora o comportamento de autogerenciamento^(24)^.

Outro dado importante apontado no estudo foi que pacientes que não fazem uso de psicotrópicos mostraram maior aderência ao tratamento medicamentoso. Isso sugere que o estado mental e emocional do paciente pode influenciar sua capacidade de seguir corretamente o tratamento^(25)^. Dessa forma, é importante que os enfermeiros se atentem a essas questões e, se necessário, envolvam profissionais de saúde mental no cuidado dos pacientes que precisam de um suporte adicional^(26)^.

Em conjunto, esses resultados destacam a complexidade do autocuidado com o DM, indicando a necessidade de abordagens personalizadas e educativas ao longo do tratamento.

Os resultados apresentados têm implicações práticas para profissionais de saúde, que devem adotar uma abordagem educativa, acessível e proativa para melhorar a adesão dos pacientes ao autocuidado. Isso inclui o uso de tecnologias adaptadas para diferentes públicos, a busca ativa por pacientes com menor frequência de consultas e estratégias de ensino que tornem o tratamento mais compreensível e viável para todos. Essas medidas são essenciais para prevenir complicações como a DPDM e melhorar a qualidade de vida das pessoas com DM.

Embora algumas variáveis tenham demonstrado associação estatisticamente significativa com os escores do QAD, os tamanhos de efeito observados foram pequenos (r<0,50), o que sugere que tais associações podem não ter grande relevância clínica. Neste sentido, os fatores identificados podem explicar uma fração da variabilidade nos escores de adesão ao autocuidado. Portanto, é importante interpretar os resultados com cautela e considerar análises futuras que incluam outras variáveis contextuais, comportamentais ou psicossociais que possam contribuir com maior magnitude para a compreensão da adesão ao autocuidado com o DM.

### Limitações do estudo

Entre as limitações do estudo, estão o fato de ser transversal, o que não permite estabelecer uma relação de causa e efeito entre a adesão ao autocuidado e as variáveis explicativas, principalmente em relação ao risco de ulceração; a seleção de participantes apenas da ESF da zona urbana, excluindo-se os demais espaços onde pacientes com DM são atendidos na APS; e a relativa generalização dos resultados, pois, apesar de o município de coleta ter um número populacional considerável, não é possível estendê-los a outros municípios brasileiros.

Outra limitação do estudo refere-se à ausência de variáveis contextuais e psicossociais, como rede de apoio, acesso aos serviços de saúde e suporte familiar, que influenciam diretamente a adesão ao autocuidado. Além disso, não foram incluídas avaliações específicas da cognição ou do estado emocional dos participantes, o que restringe a interpretação de achados como o uso de psicotrópicos.

### Contribuições para as áreas da enfermagem e saúde

O estudo aponta achados que alertam sobre o DM e a necessidade do acompanhamento das pessoas na APS ser realizado com a inclusão de um olhar para as pernas e pés. Há muitos anos, existem manuais e orientações sobre a APS, com destaque para a ESF como porta de entrada e responsável pelo controle do DM, incluindo a prevenção de complicações e/ou sua detecção precoce. Contudo, o aumento de ulcerações e amputações como resultado da DPDM despertam preocupação sobre esse problema de saúde pública^(4,27)^. O enfermeiro pode ser um dos principais profissionais para o rastreamento das ulcerações nos pés de pessoas com DM.

## CONCLUSÕES

As análises indicaram que fatores clínicos, como tempo de diagnóstico, número de consultas e polifarmácia, influenciam os escores de autocuidado. No entanto, os tamanhos de efeito reduzidos sugerem que variáveis sociodemográficas e contextuais, como suporte emocional, rede de apoio e acesso aos serviços, também exercem papel relevante e merecem ser exploradas em estudos futuros.

O estudo identificou que quase metade da amostra apresentou risco para ulceração nos pés segundo critérios clínicos de PSP e/ou DAP, o que reforça a necessidade de ações preventivas mais eficazes na APS. Além disso, a adesão ao autocuidado entre pessoas com DM na APS permanece centrada em práticas biomédicas, como uso de medicamentos e monitorização glicêmica, enquanto atividades que exigem mudança de comportamento, como cuidados com os pés, alimentação e atividade física, apresentando baixa adesão.

Nesse sentido, torna-se urgente o fortalecimento das ações multiprofissionais na APS, com ênfase em abordagens educativas, contínuas e personalizadas, capazes de promover a autonomia do paciente e a integração do cuidado com foco na prevenção de complicações, como a DPDM.

## Data Availability

Os dados de pesquisa só estão disponíveis mediante solicitação.
